# Vaccine hesitancy in the French population in 2016, and its association with vaccine uptake and perceived vaccine risk–benefit balance

**DOI:** 10.2807/1560-7917.ES.2018.23.17.17-00816

**Published:** 2018-04-26

**Authors:** Dominique Rey, Lisa Fressard, Sébastien Cortaredona, Aurélie Bocquier, Arnaud Gautier, Patrick Peretti-Watel, Pierre Verger, on behalf of the Baromètre santé 2016 group

**Affiliations:** 1 ORS PACA, Southeastern Health Regional Observatory, Marseille, France; 2 INSERM, UMR S912, « Economics and Social Sciences Applied to Health & Analysis of Medical Information » (SESSTIM), Marseille, France; 3 Aix-Marseille University, IRD, UMR-S912, Marseille, France; 4 IRD, AP-HM, VITROME, IHU-Méditerranée Infection, Aix-Marseille University, Marseille, France; 5 Santé Publique France, French National Public Health Agency, Saint-Maurice, France; 6 Baromètre santé 2016 group

**Keywords:** vaccine hesitancy, human papillomavirus vaccine, seasonal influenza vaccine, measles vaccine, hepatitis B vaccine

## Abstract

Vaccine hesitancy (VH) is prominent in France. **Objectives:** This study aimed to estimate the prevalence and socio-demographic correlates of VH in sub-groups of the French population and to investigate the association of VH with both vaccine uptake and perceived risk–benefit balance (RBB) for four vaccines. **Methods**: During the 2016 Health Barometer – a national cross-sectional telephone survey in a representative sample of the French population – parents of 1–15 year-old children, parents of 11–15 year-old girls and elderly people aged 65–75 years were asked about VH (using three questions adapted from the World Health Organization definition), vaccine uptake and perceived RBB for measles and hepatitis B (children’s parents), human papillomavirus (girls’ parents) and seasonal influenza (elderly people) vaccines. **Results**: A total of 3,938 parents including 959 girls' parents – and 2,418 elderly people were interviewed. VH prevalence estimates were 46% (95% confidence interval (CI): 44–48) among parents, 48% (95%CI: 45–51) among girls’ parents and 35% (95% CI: 33–36) among elderly people, with higher estimates associated with high education level, children’s age (10–15 years), and, for the elderly, poor perception of health status. VH was associated with uncertainty about and/or an unfavourable perception of vaccines’ RBB for the four vaccines and with lower self-reported vaccine uptake, except for human papillomavirus vaccine in girls. Results were confirmed by multivariable analysis. **Conclusion**: Further research is needed to study the association between VH and vaccine uptake for other vaccines, and to design and validate measurement tools to monitor VH over time.

## Introduction

While vaccination is globally accepted, ‘vaccine hesitancy’ (VH) is a notion increasingly invoked to refer to the reluctance to various vaccines observed in many countries [1]. The World Health Organization’s Strategic Advisory Group of Experts (SAGE) on Immunization provides the following definition of VH: ‘VH refers to delay in acceptance or refusal of vaccines despite availability of vaccination services. VH is complex and context specific, varying across time, place and vaccines. It is influenced by factors such as complacency, convenience and confidence.*’* [2]. VH depends on many factors including (i) contextual factors, (ii) individual and group influences (e.g. people’s trust in health authorities and in healthcare workers; self-perception of health), and (iii) perceived risks associated with vaccine-preventable diseases and trust in their corresponding vaccines [3,4]. The last 20 years in France have seen continued controversy about vaccines: unfavourable opinions towards vaccination in general have increased [5] and a study in 2015 involving 67 countries found that, among these, the French population had the least confidence in vaccine safety [1].

To identify vaccine concerns early, quantify their prevalence and evaluate the impact of strategies designed to address these concerns, measuring VH and following its evolution over time is essential. However, with the exception of the 18-item Parent Attitudes about Childhood Vaccines (PACV) scale, designed by Opel [6] in 2011 to identify vaccine-hesitant parents, and an eight-item vaccine confidence scale designed to identify parents at risk of refusing adolescent vaccines [7], concise tools for measuring VH prevalence in various groups in the general population and data on VH prevalence are lacking.

The Health Barometer (Baromètre santé) 2016 is the eighth in a series of French national cross-sectional surveys addressing health issues in representative population samples. It was designed and conducted by the French Public Health Agency (Santé publique France). One section of the questionnaire of the 2016 survey concerned vaccination issues. The goals of this study were (i) to estimate the prevalence of VH in parents of children, parents of adolescent girls and elderly people, (ii) to determine VH’s socio-demographic correlates, (iii) to both understand in each concerned sub-group, which specific vaccine-related behaviours and perceptions VH captures, and to study associations between VH and both self-reported behaviours and the perceived risk–benefit balance (RBB) for the measles, hepatitis B (HBV), human papillomavirus (HPV), and seasonal influenza (SI) vaccines.

## Methods

### Population

A Computer Assisted Telephone Interview (CATI) survey took place between January and July 2016 in mainland France, and was conducted in a representative sample of French people aged 15–75 years. To adequately cover the population, an overlapping dual-frame design of landline and mobile phone numbers was used [8]. Telephone numbers were generated randomly from the prefixes allocated by the Electronic Communications and Postal Regulatory Authority (ARCEP). All households with at least one French-speaking individual aged 15–75 years were eligible. In each household, one respondent was selected at random from eligible household members for landline phones, or from eligible regular mobile users for mobile phones. The French National Commission for Computer Data and Individual Freedom (CNIL) approved the survey.

The vaccination section of the 2016 survey was designed to evaluate perceptions and behaviours in three population sub-groups: parents of children aged 1–15 years (PC), parents of adolescent girls aged 11–15 years (PAG) and individuals aged 65–75 years (EP). The PAG group was part of the PC group, and therefore analysis of the latter included the former. Persons in these three sub-groups were asked questions about four vaccine(s) recommended in the French official vaccine schedule: the HBV vaccine is recommended for all infants, and as a catch-up strategy for children up to 15 years [9]; the HPV vaccine is recommended for girls aged 11–14 years, and also as a catch-up strategy for girls aged 15–19 years who have not initiated the vaccination [9]; the measles vaccine is recommended for all infants and for people born after 1980 who have never been vaccinated [9]; the SI vaccine is recommended for all individuals 65 years-old or over [9]. These four vaccines have a non-optimal coverage in their target groups (90% for HBV at 24 months of age in 2016, 20% for one dose HPV in 2015, 80% for two doses of measles in 2016, and 51% for SI in the elderly during the 2015/16 season [10]) with respect to French public health objectives (95% for all vaccines, except 75% for SI in the elderly).

### Questionnaire

The questionnaire included three questions adapted from the SAGE group’s definition of VH: (i) ‘Have you ever refused, (for your child (PC/PAG)/for yourself (EP)), a vaccine recommended by your physician, because you considered this vaccination dangerous or useless?’ (ii) ‘Have you ever delayed a vaccine recommended by your physician, (for your child (PC/PAG)/for yourself (EP)), because you hesitated over it?’ and (iii) ‘Have you ever had a vaccine, (for your child (PC/PAG)/for yourself (EP)), despite having doubts about its effectiveness?’ (with possible answers being: ‘Yes’, ‘No’, ‘Does not know’). Notions of effectiveness and safety were included in the wording of the questions to rule out reasons for vaccine delay, which were not related to doubt or opposition to vaccines (e.g. child having a cold, forgetting a vaccine appointment) [11]. In each sub-group, people were asked questions about the vaccine(s) specifically recommended to them or their children. Moreover, for each vaccine studied, five other questions were asked. Of these, one question was about children’s immunisation status (PC/PAG) and own immunisation status (EP), and four were about perceptions of the frequency and severity of the disease, and the effectiveness and risks associated with the corresponding vaccine (‘Do you think: the disease is serious? the disease is frequent? the vaccine is effective? the vaccine may cause severe side effects?*’*) (with possible answers being: ‘Yes, absolutely’/‘Somewhat’/’Not really’/’Not at all’). Questions on perceived frequency and severity of HPV infection, and perceived risks and effectiveness of the corresponding vaccine were only asked to PAG who reported they had already heard about the HPV vaccine or a vaccine to prevent cervical cancer. Finally, the questionnaire also collected information on socio-demographic characteristics of participants, and their self-perception of health status (five modalities from ‘excellent to very poor’). We used the equivalised household income per month, which takes into account household size and composition, to estimate standard of living [12].

### Statistical analysis

Data were weighted taking into account the numbers of eligible people and telephone lines in the household and socio-demographic data for the French population (sex, age, education level, number of people in household, size of town of residence, and region of residence).

We constructed the variable *‘Vaccine hesitancy’* (yes/no) to classify people who had refused and/or delayed and/or accepted despite doubts on vaccination (study goal 1). Those who answered ‘no’ to the three questions were considered not vaccine hesitant, as were the 0.3% who answered ‘does not know’.

The four questions about perceptions of the studied diseases and their corresponding vaccines were combined to build a ‘*perceived RBB*’ variable for each vaccine. To do this, responses were first dichotomised as follows: ‘Absolutely’/’Somewhat’ versus ‘Not really’/’Not at all’. Second, four categories were created: (i) very favourable perception of vaccine RBB (perception that the disease is serious or frequent, and the vaccine is effective and safe); (ii) no perceived risk (perception that the disease is not serious and rare, and the vaccine is effective and safe); (iii) uncertainty about vaccine RBB (perception that the disease is serious and frequent, and the vaccine is ineffective or unsafe); (iv) unfavourable perception of vaccine RBB (perception that the disease is not serious or rare, and the vaccine is ineffective or unsafe).

#### Statistical analyses

All analyses were carried out separately among each of the three sub-groups. To study socio-demographic correlates of VH (study goal 2), we implemented simple and multiple logistic regression analyses, the latter including all the studied socio-demographic variables. For study goal 3, we defined the following eight dependent variables: parents self-reported vaccination uptake for their children for measles and HBV (PC) and HPV (PAG), and EP self-reported vaccination uptake for SI in 2015/16, as well as perceived RBB for all relevant vaccines. We studied associations with VH for each of these dependent variables separately by first carrying out bivariate analyses and chi-squared tests, and then fitting a multiple logistic regression adjusted for the following socio-demographic variables (age, sex, education level, household income, number and age of children in the household) and perceived health status in EP. With regard to the perceived RBB dependent variables, we used multinomial logistic regressions because they included > 2 categories (see Table 3 in the results): the ‘no risk perceived’ category, which accounted for only ≤ 2% of participants, was aggregated with the ‘very favourable perception of vaccine RBB’ category (as they had the same perceptions regarding the safety and effectiveness of the vaccine, and same VH distribution). We computed the variance inflation factor (VIF) to test for multicollinearity in equivalent linear models and interpreted VIF values < 5 as presenting no multicollinearity issues [13]. The Hosmer–Lemeshow test and its generalised version were performed to assess the goodness-of-fit of the logistic and multinomial logistic models, respectively [14]. All analyses were based on two-sided p values, with statistical significance defined by p ≤ 0.05. They were conducted with SAS 9.4 statistical software (SAS Institute, Cary, NC).

## Results

Fifty-two per cent and 48% responded, respectively, to the sample of landline and mobile phones for Health Barometer 2016. The VH questions were only proposed to three population sub-groups of the 15,213 people interviewed: 3,938 PC – including 959 PAG – and 2,418 EP. Mean ages were 39.5 (standard deviation (SD): 7.8); 43.6 (SD: 6.1); and 69 (SD: 2.8) years, respectively. There were more women than men in all three groups (56.3% among PC, 57.3% among PAG, and 53.2% among EP).

### Vaccine hesitancy prevalence and associated socio-demographic characteristics

The prevalence of VH among PC and PAG regarding vaccination of their children was 46% and 48%, respectively; it was lowest in parents of children aged 0–9 years (43%) (Table 1). Prevalence in EP regarding their own vaccination reached 35% (Table 1). Table 2 details the socio-demographic characteristics of the three sub-groups. In multiple logistic regressions, VH was significantly associated with a higher level of education irrespective of the population sub-group, a low income in PAG and a poor or very poor self-perceived health in the EP (Table 2). VH was also significantly more frequent among women than men. Finally, PC with at least one child aged 10–15 years were more likely to be vaccine hesitant than parents with younger children.

**Table 1:** Prevalence of vaccine hesitancy according to the WHO SAGE definition in population sub-groups, Health Barometer, France 2016, (n = 6,356^a^)

Questions adapted from the WHO SAGE group’s definition of vaccine hesitancy	Parents of children aged 1–15 years (n = 3,938)		Parents of girls aged 11–15 years (n = 959) % (95% CI)	65–75 year-olds (n = 2,418) % (95% CI)
	1–9 years (n = 1,811) % (95% CI)	10–15 years (n = 2,127) % (95% CI)		
Has refused a vaccine recommended by their physician they considered dangerous or useless (yes)	22.8 (20.8–24.7)	29.4 (27.5–31.4)	29.3 (26.4–32.1)	16.1 (14.7–17.6)
Has delayed a vaccine recommended by their physician because of doubts about it (yes)	15.3 (13.6–16.9)	18.1 (16.5–19.8)	18.9 (16.4–21.4)	15.9 (14.5–17.4)
Has had a vaccine despite doubts about its efficacy (yes)	26.7 (24.6–28.7)	27.0 (25.2–28.9)	26.9 (24.1–29.7)	19.1 (17.6–20.7)
Vaccine hesitancy (defined as a ‘yes’ response to at least one of these three questions)	42.9 (40.6–45.2)	48.5 (46.3–50.6)	48.2 (45.1–51.4)	34.5 (32.6–36.4)

CI: confidence interval; WHO SAGE: World Health Organization’s Strategic Advisory Group of Experts on Immunization.

^a^ The sum of the three groups exceeds the total of the sample as parents of 11–15 year-old girls are part of the group of parents of children aged 1–15 years.

**Table 2:** Simple and multiple logistic regression analyses of socio-demographic characteristics of three sub-groups of people and their association with vaccine hesitancy, Health Barometer, France, 2016 (n = 6,356^a^)

Group and characteristics	Total		Vaccine hesitant (ref. No)			
	N	Weighted %	N	Weighted %	OR (95%CI)	aOR (95%CI)
**Parents of children aged 1–15 years**	**3,938**	**100**	**1,927**	**45.9**	**NA**	**NA**
**Sex**						
Men	1,656	43.7	727	39.4	1	1
Women	2,282	56.3	1,200	60.6	**1.4 (1.2–1.6)**	**1.3 (1.2–1.5)**
**Age in years**						
19–34	917	26.8	428	25.9	1	1
35–45	1,933	47.9	967	48.5	1.1 (0.9–1.3)	0.9 (0.8–1.1)
46–73	1,088	25.3	532	25.6	1.1 (0.9–1.3)	1.0 (0.8–1.2)
**Educational level^b^**						
Lower than high school	1,048	43.6	442	37.8	1	1
High school	814	19.3	394	20.2	**1.4 (1.2–1.7)**	**1.4 (1.2–1.7)**
Bachelor’s Degree	1,201	22.3	630	25.6	**1.7 (1.4–2.0)**	**1.7 (1.4–2.0)**
Master's Degree or higher	872	14.8	459	16.4	**1.6 (1.3–1.9)**	**1.7 (1.4–2.2)**
**Household income (Euros per month)**						
≤ 833	681	26.6	323	25.0	1	1
833–1,250	881	24.5	438	24.3	1.1 (0.9–1.3)	1.1 (0.9–1.3)
1,250–1,785	1,331	28.6	658	29.6	**1.2 (1.0–1.4)**	1.0 (0.8–1.2)
≥ 1,785	1,045	20.4	508	21.2	**1.2 (1.0–1.5)**	1.0 (0.8–1.2)
**Age group of oldest child in years (among all children aged 1–15 years)**						
0–9	1,811	45.7	818	42.7	1	1
10–15	2,127	54.3	1,109	57.3	**1.3 (1.1–1.4)**	**1.4 (1.1–1.6)**
**Number of children aged 1–15 years**						
1	1,794	47.4	857	46.9	1	1
>1	2,144	52.6	1,070	53.2	1.0 (0.9–1.2)	1.0 (0.8–1.2)
**Parents of girls aged 11–15 years**	**959**	**100**	**504**	**48.2**	**NA**	**NA**
**Sex**						
Men	389	42.7	175	36.7	1	1
Women	570	57.3	329	63.3	**1.6 (1.3–2.1)**	**1.6 (1.3–2.1)**
**Age in years**						
26–40	236	28.6	126	28.1	1	1
41–46	435	44.5	237	45.2	1.1 (0.8–1.4)	1.0 (0.7–1.3)
47–72	288	26.9	141	26.6	1.0 (0.7–1.4)	1.0 (0.7–1.4)
**Educational level^c^**						
Lower than high school	298	50.2	119	39.4	1	1
High school	185	16.9	103	19.7	**2.1 (1.5–3.0)**	**2.0 (1.4–2.9)**
Bachelor’s Degree	281	20.6	167	25.9	**2.5 (1.8–3.5)**	**2.3 (1.6–3.4)**
Master's Degree or higher	194	12.3	114	15.0	**2.3 (1.6–3.5)**	**2.4 (1.5–4.0)**
**Household income (Euros per month)**						
≤ 833	337	28.9	76	23.5	1	1
833–1,250	314	27.8	131	30.2	**1.7 (1.2–2.4)**	**1.6 (1.1–2.3)**
1,250–1,785	171	24.4	160	25.1	**1.5 (1.1–2.2)**	1.1 (0.7–1.6)
≥ 1,785	137	18.9	137	21.3	**1.8 (1.3–2.7)**	1.2 (0.8–1.9)
**Number of children aged 1–15 years**						
1	580	62.7	193	38.8	1	1
> 1	379	37.3	311	61.2	**1.2 (1.0–1.6)**	1.2 (0.9–1.6)
**65–75 years-olds**	**2,418**	**100**	**839**	**34.5**	**NA**	**NA**
**Sex**						
Men	992	46.8	321	43.5	1	1
Women	1,426	53.2	518	56.5	**1.2 (1.0–1.5)**	**1.2 (1.0–1.4)**
**Age in years**						
65–69	1,425	60.2	504	60.8	1	1
70–75	993	39.8	335	39.2	1.0 (0.8–1.1)	1.0 (0.8–1.1)
**Educational level^d^**						
Lower than high school	1,261	67.5	416	65.5	1	1
High school	416	13.4	149	13.5	1.1 (0.8–1.4)	1.1 (0.8–1.5)
Bachelor’s Degree	388	10.7	150	12.9	**1.4 (1.1–1.8)**	**1.5 (1.1–2.0)**
Master's Degree or higher	346	8.4	119	8.1	1.0 (0.7–1.3)	1.0 (0.8–1.6)
**Household income (Euros per month)**						
≤ 1,166	483	26.1	173	26.5	1	1
1,167–1,749	524	25.4	179	25.0	1.0 (0.8–1.2)	1.0 (0.8–1.3)
1,750–2,499	626	22.1	225	22.9	1.0 (0.8–1.3)	1.0 (0.8 –1.3)
≥ 2,500	785	26.4	262	25.6	0.9 (0.7–1.2)	0.9 (0.7–1.2)
**Perceived health**						
Very good excellent	502	19.0	162	17.1	1	1
Good	1,520	62.9	518	61.2	1.3 (0.9–1.4)	1.2 (0.9–1.5)
Poor/very poor	396	18.0	159	21.8	**1.6 (1.2–2.1)**	**1.7 (1.3–2.2)**

aOR: adjusted odds ratio; CI: confidence interval; NA: non applicable; OR: odds ratio.

aOR and ORs in bold are statistically different from 1 at the p < 0.05 level.

Hosmer–Lemeshow goodness-of-fit test: p > 0.05.

^a^ The sum of the three groups exceeds the total of the sample as parents of 11–15 year-old girls are part of the group of parents of children aged 1–15 years.

^b^ Three missing values.

^c^ One missing value.

^d^ Seven missing values.

### Association between vaccine hesitancy and perceived risk–benefit balance of the vaccines

The most favourably perceived vaccine by parents (PC and PAG) was for measles (45% had a very favourable perception of the vaccine’s RBB) (Table 3). A quarter of parents had an unfavourable perception of vaccines’ RBB. For HBV, HPV and SIV, more than 60% of parents and EP considered the vaccines as not effective or not safe (unfavourable perception or uncertainty about RBB). In bivariate analyses, perceived vaccine RBB was always more often unfavourable in vaccine hesitant individuals than in others, irrespective of the subgroup and vaccine. PAG was the only subgroup where the proportion of individuals uncertain about the HPV vaccine’s RBB was higher among non-vaccine hesitant parents than among hesitant ones, but this difference was not significant. The multivariable analysis in the three population sub-groups for the four studied vaccines, showed that vaccine hesitant people were more likely than non-vaccine hesitant ones to be uncertain about or unfavourably perceive the RBB (Table 4). We found no issue of multicollinearity.

**Table 3:** Self-reported behaviours and perceived risk–benefit balances for four vaccines, according to whether population sub-groups were vaccine hesitant or not, Health Barometer, France, 2016 (n = 6,356^a^)

Behaviours	Parents of children aged 1–15 years n = 3,938				Parents of girls aged 11–15 years n = 959				65–75 year-olds n = 2,418			
	Weighted %			p value^b^	Weighted %			p value^b^	Weighted %			p value^b^
	All (100%)	Vaccine hesitant			All (100%)	Vaccine hesitant			All (100%)	Vaccine hesitant		
		Yes (46%)	No (54%)			Yes (48%)	No (52%)			Yes (35%)	No (65%)	
**Immunisation status**												
Has at least one child/one daughter												
Vaccinated against HBV	49.0	42.1	54.8	≤ 0.001	NA	NA	NA	NA	NA	NA	NA	NA
Vaccinated against measles	92.9	91.8	93.9	≤ 0.01	NA	NA	NA	NA	NA	NA	NA	NA
Vaccinated against HPV	NA	NA	NA	NA	16.9	16.7	17.0	NS	NA	NA	NA	NA
Vaccinated against SI in 2015/16	NA	NA	NA	NA	NA	NA	NA	NA	45.9	37.8	50.1	≤ 0.001
**Perceptions of the vaccines risk**–**benefit balance**												
**HBV vaccine^c^**												
Unfavourable balance	26.2	33.8	19.6	≤ 0.001	NA	NA	NA	NA	NA	NA	NA	NA
Uncertain balance	36.5	40.1	33.3		NA	NA	NA	NA	NA	NA	NA	NA
No risk perceived	0.7	0.5	0.8		NA	NA	NA	NA	NA	NA	NA	NA
Very favourable balance	36.7	25.6	46.2		NA	NA	NA	NA	NA	NA	NA	NA
**Measles vaccine^d^**												
Unfavourable balance	26.8	32.6	21.8	≤ 0.001	NA	NA	NA	NA	NA	NA	NA	NA
Uncertain balance	21.0	21.4	20.6		NA	NA	NA	NA	NA	NA	NA	NA
No risk perceived	7.6	6.4	8.6		NA	NA	NA	NA	NA	NA	NA	NA
Very favourable balance	44.7	39.6	49.0		NA	NA	NA	NA	NA	NA	NA	NA
**HPV vaccine^e^**												
Unfavourable balance	NA	NA	NA	NA	24.2	32.8	15.6	≤ 0.001	NA	NA	NA	NA
Uncertain balance	NA	NA	NA	NA	37.7	36.4	39.1		NA	NA	NA	NA
No risk perceived	NA	NA	NA	NA	2.1	2.0	2.3		NA	NA	NA	NA
Very favourable balance	NA	NA	NA	NA	35.9	28.8	43.0		NA	NA	NA	NA
**Seasonal influenza vaccine^f^**												
Unfavourable balance	NA	NA	NA	NA	NA	NA	NA	NA	16.3	19.1	14.7	≤ 0.001
Uncertain balance	NA	NA	NA	NA	NA	NA	NA	NA	44.5	54.2	39.2	
No risk perceived	NA	NA	NA	NA	NA	NA	NA	NA	0.7	0.7	0.7	
Very favourable balance	NA	NA	NA	NA	NA	NA	NA	NA	38.6	26.0	45.5	

HBV: hepatitis B virus; HPV: human papillomavirus; NA: non applicable; NS: not significant; SI: seasonal influenza.

^a^ The sum of the three groups exceeds the total of the sample as parents of girls aged 11–15 years are part of the group of parents of children aged 1–15 years.

^b^ Chi-squared p value.

^c^ 154 missing values due to ‘does not know’ (DNK) answers to questions on disease's seriousness/frequency or on vaccine's safety/side effects.

^d^ 208 missing values.

^e^ 135 parents excluded because they were not aware of the vaccine, 54 missing values.

^f^ 60 missing values.

**Table 4:** Results from multiple multinomial logistic regressions to assess association between unfavourable or uncertain perceptions of four vaccines’ risk–benefit balances and vaccine hesitancy, Health Barometer, France, 2016 (n = 6,356)

Vaccine hesitancy	HBV		Measles		HPV		SI	
	(n = 3,781 parents of children aged 1–15 years)^a^		(n = 3,727 parents of children aged 1–15 years)^b^		(n = 769 parents of girls aged 11–15 years^)c^		(n = 2,351 65–75 year-olds)^d^	
	Perception of the vaccine RBB^e^		Perception of the vaccine RBB^e^		Perception of the vaccine RBB^e^		Perception of the vaccine RBB^e^	
	Unfavourable	Uncertain	Unfavourable	Uncertain	Unfavourable	Uncertain	Unfavourable	Uncertain
	n = 1,075	n = 1,287	n = 942	n = 709	n = 182	n = 267	n = 374	n = 956
	aOR (95%CI)^f^		aOR (95%CI)^f^		aOR (95%CI)^g^		aOR (95%CI)^h^	
No (reference)	1	1	1	1	1	1	1	1
Yes	**3.1 (2.6–3.7)**	**2.3 (2.0–2.7)**	**2.1 (1.8–2.5)**	**1.5 (1.2–1.8)**	**3.6 (2.4–5.4)**	**1.6 (1.1–2.3)**	**2.4 (1.8–3.1)**	**2.5 (2.0–3.0)**

aOR (95%CI): adjusted odds-ratio (95% confidence interval); HBV: hepatitis B virus; HPV: human papillomavirus; RBB: risk–benefit balance; SI: seasonal influenza.

aOR in bold are significantly different from 1. Generalised Hosmer–Lemeshow goodness-of-fit tests for HBV/Measles/HPV/SI models: p > 0.05.

^a^ 154 missing values for dependent variable and three missing values for educational level: 3,781 parents with complete data.

^b^ 208 missing values for the dependent variable and three missing values for educational level: 3,727 parents with complete data.

^c^ 189 missing values for the dependent variable and one for educational level: 769 parents with complete data.

^d^ 60 missing values for the dependent variable and seven missing values for educational level: 2,351 elderly with complete data.

^e^ Reference: very favourable or no risk perceived.

^f^ Odds ratios adjusted for age, sex, educational level, income, number and age of children – see Table 2 for the detailed list of adjustment variables.

^g^ Odds ratios adjusted for age, sex, educational level, income, number of children – see Table 2 for the detailed list of adjustment variables.

^h^ Odds ratios adjusted for age, sex, educational level, income and perceived health – see Table 2 for the detailed list of adjustment variables.

### Associations between vaccine hesitancy and self-reported vaccination uptake

In the PC group, self-reported vaccination uptake for children was 49% for HBV and 93% for measles. Seventeen per cent of PAG reported that at least one of their daughters had been vaccinated against HPV. Forty-six per cent of EP reported SI vaccination for themselves during the 2015/16 winter (Table 3). Vaccine hesitant PC were significantly less likely than non-hesitant ones to report HBV and measles vaccination for their children. We found a similar result in EP for SI vaccination for themselves. In contrast, VH in PAG was not associated with self-reported vaccine uptake of the HPV vaccine (Table 3). Multivariable analyses adjusted for socio-demographic characteristics confirmed these results (Table 5). We found no issue of multicollinearity.

**Table 5:** Results from multiple logistic regressions assessing association between self-reported vaccine uptake and vaccine hesitancy, Health Barometer, France, 2016 (n = 6,356)

Vaccine hesitancy	Parent has had at least one child^a^ vaccinated against			Person has vaccinated themselves^a^ against
	HBV	Measles	HPV	SI
	(n = 3,935 parents of children aged 1–15 years)^b^		(n = 958 parents of girls aged 11–15 years)^c^	(n = 2,411 65–75 year-olds)^d^
	aOR (95%CI)^e^	aOR (95%CI)^e^	aOR (95%CI)^e^	aOR (95%CI)^f^
No (reference)	1	1	1	1
Yes	**0.6 (0.5–0.7)**	**0.6 (0.5–0.8)**	0.9 (0.6–1.3)	**0.6 (0.5–0.7)**

aOR (95%CI): adjusted odds-ratio (95% confidence interval); HBV: hepatitis B virus; HPV: human papillomavirus; SI: seasonal influenza.

aOR significantly different from 1 are in bold. Hosmer–Lemeshow goodness-of-fit tests for HBV/Measles/HPV/SI models: p > 0.05.

^a^ Reference: parent had no child vaccinated, or elderly person did not get vaccinated.

^b^ Three missing values for educational level.

^c^ One missing value for educational level.

^d^ Seven missing values for educational level.

^e^ Odds ratios adjusted for age, sex, educational level, income, number and age of children – see Table 2 for the detailed list of adjustment variables.

^f^ Odds ratios adjusted for age, sex, educational level, income and perceived health – see Table 2 for the detailed list of adjustment variables.

## Discussion

This survey provides estimations of VH prevalence according to the definition proposed by the SAGE group [2] in large sub-groups of the French population. Our results showed that VH prevalence was widespread among parents of children (PC) aged 1–15 years-old (46%), particularly parents of adolescent girls (PAG) aged 11–15 years old) (48%), and especially among those with a high education level (Table 2). VH was associated with uncertainty about and/or an unfavourable perception of RBB for the four vaccines studied (measles, HPV, HBV, and SI), while associations between VH and self-reported vaccination uptake were weaker and not systematic.

Our VH prevalence estimations reflect some of the highest values published to date (from 12 to 40%) [6,11,15], but differences between studies in terms of VH definitions and populations studied make comparisons difficult [1]. The percentage of parents who had refused a vaccine for their children in our study (22.8 to 29.4% according to the age of the child) was higher than what is usually reported (from 6 to 28%) [11,15,16]. Unlike some other studies however, our estimation of VH was based on generalised questions and not restricted to particular vaccines. Furthermore, interviewed parents had children belonging to large age groups including adolescents. Moreover, our questions referred to the children’s lifetime and opportunities to refuse a vaccine increase over time. VH was however, less frequent in EP than in PC and PAG. This may be linked to possible recall biases or to a generational phenomenon, the lack of confidence in vaccines being a relatively recent phenomenon [5].

Our findings on vaccine hesitant people (Table 2) are also in line with other studies from the United States in 2010 and 2011, which showed that parents with a high educational level were more prone to be vaccine hesitant for their children than other parents [15,17]. However, this association is not found in all studies. Indeed, some studies show an association between VH and low socio-economic status [4,17], as we observed among PAG. These contradictory results may reflect the complexity of the definition of VH that captures heterogeneous populations [4]. As in previous studies [18], we also found that mothers were more often vaccine hesitant than fathers, perhaps because they are often more involved in the medical follow-up of their children. Finally, in EP, health concerns and chronic disease have already been described as motives for attributing a low priority to or refusing influenza vaccination [19,20].

The association of VH with an unfavourable perception of RBB for the four studied vaccines mirrors other findings, showing that one of the largest areas of concern about vaccination in the general population is vaccine safety [1]. For parents, the measles vaccine had the most favourable RBB. Indeed, it is the only one of the four vaccines studied whose safety has not been the subject of national controversy in France.

In our study, associations between VH and self-reported vaccine uptake were found for all the studied vaccines, except the HPV vaccine, something also found by Roberts et al. [21] who used a modified version of the PACV. This was also the studied vaccine with the lowest self-reported vaccine uptake (only 17%). Parental reasons for vaccine refusal or doubts are specific to the HPV vaccine including: concerns regarding their adolescent daughters’ sexuality, perception that the vaccine is not needed [21], no recommendation by physician [22,23] and their belief that there is not enough reliable information on the HPV vaccine, which they still perceive as new, compared with other vaccines [21,22]. If we consider that self-reported HPV vaccine uptake was 17% in adolescent girls and that only a total of 48% of PAG were vaccine hesitant in our survey, this suggests that our VH definition, which focused on doubts about vaccine safety and effectiveness, was too restrictive to capture all parents’ HPV vaccine perceptions.

Since 2007, HPV vaccination programmes have been implemented in most European countries for the prevention of cervical cancer. A significant decrease in the frequency of high grade cervical precancerous lesions in young women has been observed in countries like Sweden and Denmark with successful HPV vaccination programmes [24]. HPV vaccination coverage rates exceed 80% in the United Kingdom and Portugal, but are still very low in some other Europeans countries, particularly in France (19% for three doses in 2015) [10]. The French government has recently extended the number of childhood compulsory vaccines from three to eleven, for all children born from 1 January 2018 [25]. The HPV vaccine is not on the list of compulsory vaccines. This fact may lead parents to the false perception that the threat from HPV infection is lower compared to other diseases and that HPV vaccination is unnecessary. It is therefore urgent to restore vaccine confidence in parents, especially in parents of adolescents, and not to limit actions to messages on vaccine safety and effectiveness, which, as suggested in our survey, are not the only components of VH in the context of HPV.

Vaccine hesitancy is also present among European vaccine providers, both for patients and for themselves [26]. Part of this comes from fear of vaccine side effects. In France, during recent controversies about the HPV vaccine, some physicians even put forward arguments against its use [27]. There is a real need to address this loss of confidence, as health professionals have a major role in their patients’ decision making about vaccination [27].

A recent review of strategies for addressing VH [28] showed that the most effective interventions were multicomponent and dialogue-based interventions, tailored to specific populations and addressing specific concerns. There is a need to improve understanding of the drivers of VH both in the population and among healthcare providers, to adapt the interventions, and to monitor VH over time once these interventions have been introduced. Large repeated surveys like the French Health Barometer could provide opportunities to measure VH in target groups on a regular basis.

### Limitations of the study

We have to acknowledge several limitations of the present study. Selection bias cannot be excluded, since approximately half of the people contacted refused to participate. However, refusals were not related to the topic of the survey, as the announcement letter did not mention vaccination. Furthermore, the response rate was similar to those observed in phone surveys carried out in large populations [29]. Moreover, our data were weighted to be representative of the studied populations. Our study shares the usual limitations of surveys based on self-reporting, an approach frequently used to assess vaccination coverage with acceptable reliability [30]. Self-reported vaccination uptake in our survey was close to the estimated vaccination coverage (VC) for influenza and measles in France, based on vaccine reimbursement data. During the 2015/16 season, VC for influenza was estimated at 51% in French people aged 65 years and over (in our survey self-reported vaccine uptake was 46%) [10]. In 2016, 95% of children aged 15 years had received one dose of the measles vaccine (93% in our survey) [10]. Self-reported HBV vaccine uptake was only 49% in children aged 1–15 years in our study although VC for HBV was estimated at 90% in 2016 in children aged 24 months [10]. The wide age range used for children in our survey may partially explain this difference, as HBV vaccine uptake in adolescents is much lower than in infants. Moreover, parents do not always know the valences contained in the hexavalent vaccine currently most used in infants in France, and they may not be aware of the specific vaccines administered. With regard to HPV, VC in 2015 was 20.4% for one dose in those aged 15 years and 19% for three doses in those aged 16 years. VC has been decreasing in these age groups since 2010 [10]. The 17% self-reported HPV vaccine uptake in our survey was probably slightly underestimated as we only considered girls aged 11–15 years even though HPV vaccine may be proposed up to 19 years as a catch-up strategy.

### Conclusion

It is essential to improve understanding of individuals considered vaccine hesitant, as they represent the first target of public health measures to improve immunisation coverage. Our short VH estimation tool, based on the SAGE definition, was easy to implement to estimate VH prevalence in sub-groups of the general population. It mostly captured perceptions about vaccines but did not enable us to systematically capture vaccine uptake behaviour, especially for HPV in adolescent girls. Further research is needed to confirm our results, to study the association between VH and vaccine uptake for other vaccines, and to design and validate measurement tools to monitor VH over time in order to help evaluate interventions implemented to address VH.
